# ConvNet and LSH-Based Visual Localization Using Localized Sequence Matching †

**DOI:** 10.3390/s19112439

**Published:** 2019-05-28

**Authors:** Yongliang Qiao, Cindy Cappelle, Yassine Ruichek, Tao Yang

**Affiliations:** 1 Australian Centre for Field Robotics (ACFR), Department of Aerospace, Mechanical and Mechatronic Engineering (AMME), The University of Sydney, Sydney, NSW 2006, Australia; 2 Connaissance et Intelligence Artificielle Distribuées (CIAD), University Bourgogne Franche-Comté, UTBM, F-90010 Belfort, France; cindy.cappelle@utbm.fr (C.C.); yassine.ruichek@utbm.fr (Y.R.); tao.yang@utbm.fr (T.Y.)

**Keywords:** visual localization, place recognition, convolutional network, sequence matching, LSH, SLAM

## Abstract

Convolutional Network (ConvNet), with its strong image representation ability, has achieved significant progress in the computer vision and robotic fields. In this paper, we propose a visual localization approach based on place recognition that combines the powerful ConvNet features and localized image sequence matching. The image distance matrix is constructed based on the cosine distance of extracted ConvNet features, and then a sequence search technique is applied on this distance matrix for the final visual recognition. To speed up the computational efficiency, the locality sensitive hashing (LSH) method is applied to achieve real-time performances with minimal accuracy degradation. We present extensive experiments on four real world data sets to evaluate each of the specific challenges in visual recognition. A comprehensive performance comparison of different ConvNet layers (each defining a level of features) considering both appearance and illumination changes is conducted. Compared with the traditional approaches based on hand-crafted features and single image matching, the proposed method shows good performances even in the presence of appearance and illumination changes.

## 1. Introduction

Visual-based vehicle localization in changing environments plays an important role in Simultaneous Localization and Mapping (SLAM) as well as the Advanced Driver Assistance Systems (ADAS) [1]. Unlike the LiDAR-based or GPS-based methods relying on expensive sensors, the vision-based localization approach using a low-cost camera is gaining popularity recently in the intelligent vehicle and robotics community [2,3]. In the vision-based localization approach, the current vehicle localization can be obtained by matching the image of the current location to an image of a previously visited position [4]. This is also known as place recognition, usually cast as an image retrieval task [5] where the current query image location is estimated using the locations of its retrieved image in a large geotagged image database. The core technique of appearance-based place recognition for visual localization is the representation of a place or location appropriately [6]. The huge appearance variations on the visual perception of a place caused by weather conditions or seasonal or illumination changes is a challenge for place-recognition-based visual localization in long-term driving.

At the early stages, place-recognition-based visual localization [7,8] mainly relies on the ad hoc features such as Scale Invariant Feature Transform (SIFT), Speeded-Up Robust Features (SURF), or GIST. Through comparing these extracted features from each image (location), the re-visit location of a vehicle or robot is determined. These traditional feature extraction techniques have made progress in visual localization.

In recent years, with the deep learning development, an automatic and powerful image feature extractor—Convolutional Network (ConvNet)—achieved a state-of-the-art performance in the computer vision and machine learning communities [9,10]. The deep ConvNet with its strong image representation ability already achieved a high-level performance on visual recognition and classification tasks [11]. With training on a large-scale image data set, ConvNet obtained discriminative and human-interpretable feature representations, the learned features were robust, and the global features could be been used for the task of visual localization without special training [12]. In long-term visual localization, two main problems need to be solved: false matching under changing environments (appearance or illumination) and the huge time cost for the high dimensional feature matching. Unlike the other deep-learning-based image recognition tasks using high dimensional features [11,13], we combine the robustness of sequence ConvNet features and the high-dimensional data reducing ability of Locality Sensitive Hashing (LSH) to develop an effective visual localization system for the long-term navigation of autonomous driving.

In this paper, a localized sequence-matching-based place recognition framework is developed. The proposed approach uses the ConvNet features and sequence matching to reduce the false recognition in long-term visual localization. In the whole visual recognition framework, ConvNet features are extracted based on a pretrained network first; then the extracted features are compared using the cosine distance. Finally, a localized sequence matching is conducted to retrieve the previous visited places based on the distance matrix. The main contributions of this paper are as follows: (1) the hierarchical nature of ConvNet features are exploited, and different ConvNet layers for place recognition under severe appearance and illumination variations are studied; (2) a comparison with state-of-the-art place recognition methods is performed on four data sets. The $F_{1}$ (the harmonic average of the precision and recall) scores attained with the conv4 layer of ConvNet for the four different data sets are higher than 0.85, which are significantly better than those of Fast Appearance Based Mapping (FAB-MAP) and Sequence Simultaneous Localisation and Mapping (SeqSLAM); (3) for real-time visual localization, a speed-up method is achieved by approximating the cosine distance between features with a hamming distance over bit vectors obtained by Locality Sensitive Hashing (LSH), by using 4096 hash bits instead of the original feature permits to accelerate by 12 times the computation time, and by keeping 95% of the original place recognition performance.

The paper is organized as follows: Section 2 briefly reviews the existed visual recognition techniques and the progress of convolutional neural networks; Section 3 describes the proposed visual localization system; the experiment platform and used data sets are illustrated in Section 4; and the experimental results are demonstrated in Section 5. In Section 6, the conclusions and plans of future works are given.

## 2. Related Works

Many approaches related to place recognition have previously been proposed in the literature in the context of visual localization systems [14,15,16]. In this section, we briefly review the current state-of-the-art place recognition method related to visual localization and the application of convolutional neural networks for various visual recognition tasks.

### 2.1. Different Representations for Place Recognition

A keypoint of place-recognition-based localization approaches is the representation or description of a “place” (location) without the influence of lighting conditions or seasons changing [17,18]. This challenging question present in many computer vision and image searching applications already leads up to numerous answers. In terms of place representation, the majority of methods can be classified into two categories: approaches based on a selective extraction of interesting or notable parts on the image (local features) and approaches based on the whole scene description (global features).

Local features, such as SIFT and SURF, have been widely used in appearance-based visual localization. The typical example is FAB-MAP [19]; it matches the appearance of the current location to a past place by converting the image into a bag-of-words representation built on local features such as SIFT or SURF. Beyond the FAB-MAP approach, SeqSLAM [20] using the sum of absolute differences (SAD) between the contrast-enhanced images [21] to measure image similarity. Then, a local-best matching over the predefined constant velocity path (i.e., the alignment between the query sequence and database sequences) is conducted to realize the place recognition under lighting and atmospheric variations. In addition, Badino et al. [22] present an outdoor visual localization approach based on a descriptor called Whole Image SURF (WI-SURF). Other local features like Local Binary Pattern (LBP) [23], Local Difference Binary (LDB) [24], and Binary Robust Independent Elementary Features (BRIEF) [25] can be also used in place recognition. However, an image description using binary descriptors may contain hundreds of local features, and features comparison is time-consuming.

In terms of global features, some researches describe the place to recognize in a holistic manner, using the whole-image (or global descriptors). A popular whole-image descriptor is GIST, which has been used for place recognition on a number of occasions [26]. Kosecka et al. [27] propose a place recognition strategy using gradient orientation histograms as image descriptors. Although global features are very fast to compute, they are not robust to effects such as lighting changes or perspective transformations [28].

Place recognition methods based on the above hand-crafted features are prone to being affected by a change in illumination or appearances. Nowadays, it is rapidly becoming apparent that, in recognition tasks, hand-crafted features are being outperformed by learned features [29]. It will be interesting and promising for visual localization to use automatically learned features thanks to the convolutional networks.

### 2.2. Convolutional Networks

Convolutional Network (ConvNet) is one of the popular deep neural networks and was firstly proposed by LeCun et al. [30] in 1989. ConvNet features are learned automatically from data sets through multi-layer supervised networks. ConvNets permit significant performance improvements on object classification or recognition to be achieved and outperform traditional hand-crafted features-based approaches [31].

Yan et al. [9] conducted a comprehensive performance comparison of the utility of features from all 21 layers for place recognition. In Reference [32], the AlexNet ConvNet model was trained on the ImageNet Large Scale Visual Recognition Challenge 2012 (ILSVRC2012) for object recognition. Sünderhauf et al. [33] presented a novel place recognition system that was built on state-of-the-art object detection methods and convolutional visual features. The astonishing power of convolutional neural network features was used to identify matching landmark proposals between images to perform place recognition over extreme appearance and viewpoint variations. The experiment results have also revealed further insights: Mid-level ConvNet features appear to be highly suitable as descriptors for landmarks of various sizes in a place recognition context.

In addition, the availability of pretrained network models makes ConvNets easy to experiment about for different tasks. It therefore appears very promising to analyze these features and to experimentally investigate their feasibility for the task of visual recognition. The software packages Overfeat [34], Caffe [35], and MatConvNet [36] provide network architectures pretrained for a variety of recognition tasks. Especially, MatConvNet, an important ConvNet MATLAB toolbox designed with an emphasis on simplicity and flexibility, allows for the fast prototyping of new ConvNet architectures and supports efficient computation on CPUs and GPUs [36].

## 3. Proposed Approach

The proposed visual localization approach can be divided into off-line and online parts. In the off-line part, a set of GPS-tagged training images $I^{train}={\left\{I_{i}^{train}\right\}}_{i=1}^{N^{train}}$ is firstly acquired, where $N^{train}$ is the number of training images. Then, the pretrained caffe-alex network (trained using the ILSVRC2012 data set) is used to extract features from training images [13]. The extracted ConvNet features from training database are noted $F^{train}$ = ${\left\{f_{i}^{train}\right\}}_{i=1}^{N^{train}}$, where $f_{i}^{train}$ is the feature extracted from the training image $I_{i}^{train}$.

For the online phase, the current testing image $I_{T}^{test}$ is input into the caffe-alex network and the ConvNet feature $f_{T}^{test}$ of the current testing image is computed. Then, $f_{T}^{test}$ is compared with the training image feature set ${\left\{f_{i}^{train}\right\}}_{i=1}^{N^{train}}$ using the cosine distance.

In terms of localized sequence matching, given a testing sequence of length $d_{s}$ (a sequence composed of images indexed from $T-d_{s}+1$ to *T*, where *T* is the index of the current image), some possible training sequence candidates are firstly determined from the training database through the ratio between the testing and training trajectory speeds. For each possible sequence candidate, a score *S* is calculated by summing all the cosine distances along each sequence. The sequence candidate that provides the minimum score can be considered as the most similar one to the testing sequence. In fact, the two best sequences (according to the matching score) are conserved to further validate the final matching result.

Following, the best matching, the candidate will be validated through a distance ratio SS (see Section 3.4). This distance ratio SS between the two minimum computed distances (corresponding to the two best candidates) is considered to validate the training sequence that finally best matches to the current testing sequence. If the ratio SS is below or equal to a threshold Th, the first best sequence candidate (with the lower matching score) is confirmed and regarded as positive; otherwise, it is considered as a negative one (in this case, no matching result is conserved). When a sequence candidate is confirmed as positive, the position can be obtained from the matched GPS-tagged training images (see Section 3.5).

**Figure 1:**
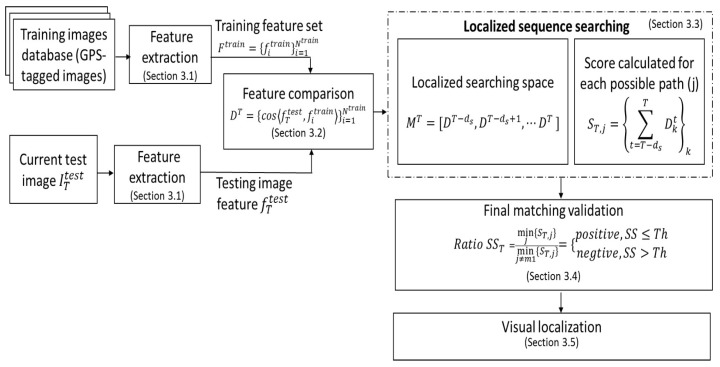
A detailed block diagram of the proposed visual localization method: Feature extraction uses a pretrained network, feature comparison uses the cosine distance, and localized sequence searching is conducted based on the potential path.

As illustrated in Figure 1, there are four important components in our visual localization approach:

- **ConvNet features extraction** (detailed in Section 3.1): ConvNet features $F^{train}$ are extracted from all training database images by off-line processing, and $f_{T}^{test}$ is extracted from the current testing image by online processing using the pretrained caffe-alex network. These learned features are robust to both appearance and illumination changes and represent each location (place) profoundly. The extracted ConvNet features will be compared in the next step.
- **Feature comparison** (detailed in Section 3.2): The cosine distances are computed between the feature $f_{T}^{test}$ of the current testing image and the features ${\left\{f_{i}^{train}\right\}}_{i=1}^{N^{train}}$ of all the images of the training database. All these distances form a vector $D^{T}$. Based on this, localized sequence matching is conducted in the next step.
- **Localized sequence matching** (detailed in Section 3.3): To achieve an efficient place recognition, localized sequence matching is used instead of single image matching. Considering the testing sequence composed of the last $d_{s}$ testing images (indexed from $T-d_{s}+1$ to *T*), localized sequence matching is conducted in the matrix $M^{T}=\left[D^{T-d_{s}+1},D^{T-d_{s}+2},\cdots ,D^{T}\right]$. According to the speed ratio between the testing and training sequences, some possible training sequence candidates in the training database can be firstly determined. A score *S* is calculated by summing all the testing image to training image cosine distances along each possible training sequence. The sequence that provides the minimum score can be considered the most similar one to the testing sequence. The two best sequence matching scores are conserved for further matching validation.
- **Final Matching Validation** (detailed in Section 3.4): The ratio SS between the two best sequence matching scores is used to verify the best sequence candidate. If the ratio SS is below or equal to a threshold Th, the first candidate (with the lower matching score) is confirmed and regarded as positive matching; otherwise, it is considered a negative one (in this case, no matching is conserved).

Several advantages of our approach can be highlighted: (1) The system uses an off-the-shelf pretrained convolutional network to extract features which makes feature extraction more convenient; (2) ConvNet features as auto-learned features are more stable and powerful. By using these robust features as descriptors for place representation, we inherit their robustness against appearance and weather changing; (3) using a localized sequence matching allows us to search in a small range rather than in the whole training database. This makes place recognition more robust and efficient.

### 3.1. ConvNet Features Extraction

In this study, a pretrained caffe-alex [35] ConvNet model and MatConvNet toolbox are deployed to extract features. The caffe-alex ConvNet model is a 21-layer network; each layer output is a deep learnt representation of the image (ConvNet feature). The low layers retain a high spatial resolution for a precise localization with low-level visual information. While high layers capture more semantic information and less fine-grained spatial details. The network is able to process images of any size equal to or greater than 227 × 227 pixels (the original caffe-alex network was trained on 227 × 227 images). Place recognition is then performed by comparing the ConvNet features extracted from the current testing image $I_{T}^{test}$ with the ConvNet features extracted from all the images ${\left\{I_{i}^{train}\right\}}_{i=1}^{N^{train}}$ of the training database.

**Figure 2:**
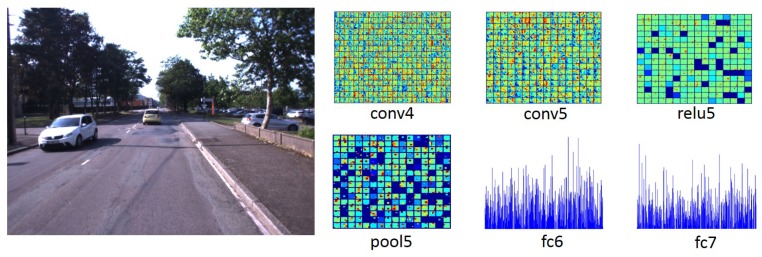
An example of scene and extracted features from different layers of the caffe-alex network. Features obtained from different ConvNet layers can serve as holistic image descriptors for place recognition.

Considering that middle layers take the advantage of the low-level and semantic information, our approach exploits feature information of these middle layers to handle large appearance changes and then alleviate false recognition. The used layers and their dimensionality are listed in Table 1. The corresponding ConvNet features generated by convolutional Networks for an example of input image are illustrated in Figure 2. It can be seen that the conv4, conv5, and relu5 layers provide more image spatial information while the pool5, fc6, and fc7 layers bring more semantic information.

### 3.2. Feature Comparison

The cosine distance is widely used to measure the feature vector similarity in the computer vision field; therefore, in our work, feature comparison is performed based on the cosine distance between the extracted ConvNet features. Each testing image feature is compared with all the images features of the training database. For that, the cosine distances between the feature $f_{T}^{test}$ of the current testing image and the features ${\left\{f_{T}^{train}\right\}}_{i=1}^{N^{train}}$ of all the images of the training database are computed as follows:

(1) $$d_{T,i}=cos\langle f_{T}^{test},f_{i}^{train}\rangle =\frac{f_{T}^{test}\cdot f_{i}^{train}}{∥f_{T}^{test}∥∥f_{i}^{train}∥};\;i=1,2,\cdots ,N^{train}$$

Then, these $N^{train}$ distances are concatenated to form a $D^{T}$ vector:

(2) $$D^{T}=\left[cos\langle f_{T}^{test},f_{1}^{train}\rangle ,cos\langle f_{T}^{test},f_{2}^{train}\rangle ,\cdots ,cos\langle f_{T}^{test},f_{N^{train}}^{train}\rangle \right]$$

where $N^{train}$ is the total number of images in the training database. $D^{T}$ is the vector that contains the cosine distance between the testing image $I_{T}^{test}$ and all the training images.

### 3.3. Localized Sequence Matching

Assume that the vehicle repeatedly travels in a route with a negligible relative acceleration or deceleration. For a given testing sequence, composed of $d_{s}$ images, indexed from $T-d_{s}+1$ to *T*, where *T* is the index of the current testing image, we search the sequence (from the training database) that corresponds to the current testing sequence. Rather than searching in the whole training database, the searching procedure is performed by considering possible training sequence candidates that are determined by the speed ratio between the training and testing sequences. This procedure is qualified as localized sequence searching.

At each time step, i.e., for each new testing image $I_{T}^{test}$, localized sequence searching is performed through a matrix $M^{T}$ constructed by cosine distance vectors $D^{t}\left(T-d_{s}+1\leq t\leq T\right)$ over the test sequence, composed of the $d_{s}$ previous images (including the current testing image):

(3) $$M^{T}=\left[D^{T-d_{s}+1},D^{T-d_{s}+2},\cdots ,D^{T}\right]$$

where $d_{s}$ is the testing sequence length (in terms of images number) that determines how far back the search goes. As defined previously, $D^{t}\left(T-d_{s}+1\leq t\leq T\right)$ is the cosine distance column vector for the current testing image $I_{t}^{test}$. It contains distances between the testing image feature $f_{t}^{test}$ and all training image features ${\left\{f_{i}^{train}\right\}}_{i=1}^{N^{train}}$.

**Figure 3:**
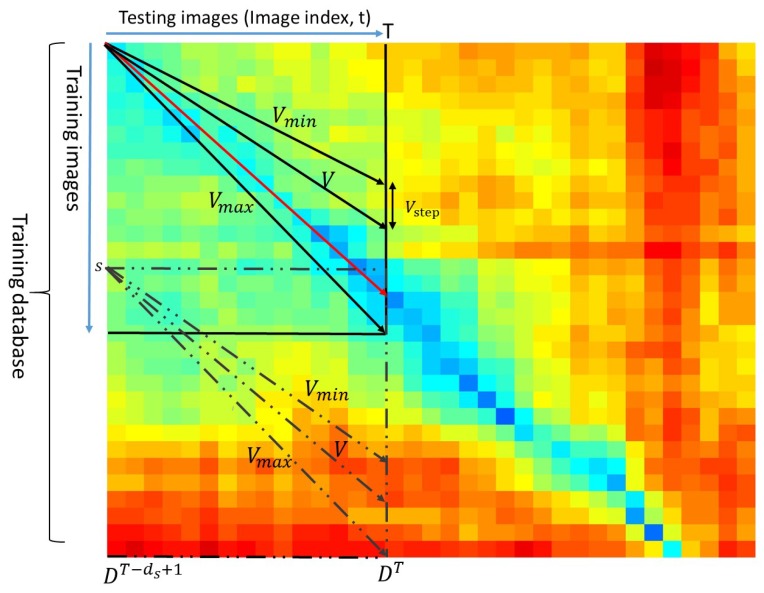
The search algorithm finds the lowest-cost straight line within the searching matrix $M^{T}$. These lines are the set of potential paths through the matrix. The red line is the lowest-cost path which aligns the testing sequence and the training sequence. Each element represents the cosine distance between two images.

For testing sequence *T* (composed of $d_{s}$ images, indexed from $T-d_{s}+1$ to *T*), due to the linear relationship restriction of the testing and training driving speed, the corresponding training sequence candidates can be firstly confirmed using the speed ratio $V(V_{min}\leq V\leq V_{max})$. As shown in Figure 3, the possible paths representing different speed ratios can be projected onto each element in the matrix $M^{T}$. Thus, the lowest-cost path which has the a minimum distance score *S* is deemed to be the best match, shown as the red line in Figure 3.

In Figure 3, each element of the matrix $M^{T}$ is the cosine distance between a testing image and a training image. A blue color in the matrix $M^{T}$ indicates a small distance value while a red color means a large distance value. Searching ranges are constrained into the space between the minimum speed ratio $V_{min}$ and the maximum speed ratio $V_{max}$. Each possible path (dark line) in the space indicates a possible match between the testing (query) sequence and the training sequence. The lowest-cost path (red line) is regarded as the best matching.

A score *S* is calculated for each path based on the distance values corresponding to the matrix components through which the straight-line passes from frame number $T-d_{s}+1$ to the current frame *T*:

(4) $$S=\sum_{t=T-d_{s}+1}^{T}D_{k(t)}^{t}$$

where k(t) is the index of the column vector $D^{t}$ by which the path (line) passes through:

(5) $$k\left(t\right)=s+V(t-\left(T-d_{s}+1\right))$$

where *s* is the training image index from which the path is originated. The initial value of *s* is 0 and then is increased by 1 at each step. *V* is the speed ratio varying between $V_{min}$, and $V_{max}$ with a step value $V_{step}$. The score *S* (sum of distance values along path (line)) is used to identify the best matching candidate (the one that has the lowest score) for each testing sequence.

### 3.4. Final Matching Validation

Given the current testing image number *T*, the corresponding testing sequence (images indexed from $T-d_{s}+1$ to *T*) can be constructed. Using the localized sequence matching method, the best two sequence candidates who have smaller scores are conserved for further validation. Suppose $S_{m1}$ and $S_{m2}$ are respectively the first and second minimum scores of the top two training sequence candidates to the testing sequence obtained by the following equation:

(6) $$\left\{\begin{array}{c} S_{T,m1}=\min_{j}\left\{S_{T,j}\right\}\\ S_{T,m2}=\min_{j(j\neq m1)}\left\{S_{T,j}\right\} \end{array}\right.$$

where *j* is the index of the training sequence candidates. In order to validate the best sequence matching, a ratio $SS_{T}$ is calculated as follows:

(7) $$SS_{T}=\frac{S_{T,m1}}{S_{T,m2}}$$

The value of ratio SS is between 0 and 1. A threshold Th is then applied to the ratio $SS_{T}$ to determine if the sequence pair (T,m1) is matched or not. If the ratio $SS_{T}$ is not larger than the threshold Th, the training sequence corresponding to m1 is matched to the current testing sequence; this is also called positive matching. Otherwise, no matching is considered (negative matching).

### 3.5. Visual Localization

After a matching result is successfully validated, the vehicle can localize itself through the matched training image position. Since the training images are tagged with GPS information, the vehicle can get its position information through the training image matched with the current testing image. This is also a topological level localization—it simply identifies the most likely location. Therefore, this is not a very accurate localization because the training and testing trajectory cannot be exactly the same.

### 3.6. Algorithm of Proposed ConvNet-Based Visual Localization

Algorithms 1 and 2 illustrate the proposed method for visual localization using localized sequence matching. It includes ConvNet feature extraction and comparison, localized sequence matching, matching validation, and visual localization steps. Algorithm 1 shows how to conduct a ConvNet feature extraction and comparison, while Algorithm 2 gives localized sequence matching methods and the final visual localization results.

**Algorithm 1** ConvNet feature extraction and comparison.**Inputs:** ${\left\{I_{i}^{train}\right\}}_{i=1}^{N^{train}}$ {training images database}; ${\left\{I_{i}^{test}\right\}}_{i=1}^{N^{test}}$ {testing images database}; $N^{train},N^{test}$ {training and testing images numbers}; **Outputs:** *D* {Cosine distance}; **Algorithm:** **for**
*i* ← 1 to $N^{test}$
**do**  **for**
*j* ← 1 to $N^{train}$
**do**   $f_{j}^{train}$ ← Feature extraction for training images;   $f_{i}^{test}$ ← Feature extraction for testing images;   $d_{i,j}$ ← cos 〈$f_{i}^{test}$, $f_{j}^{train}$〉; // Cosine distance {Section 3.2}.  **end for**  $D^{i}$ ← $\left[d_{i,1},d_{i,2},\cdots ,d_{i,N^{train}}\right]$; Column vector $D^{i}\in R^{N^{train}\times 1}$ that contains the cosine distance between the testing image $I_{i}^{test}$ and all the training images {Section 3.2}. **end for**

**Algorithm 2** Localized sequence matching and visual localization.**Inputs:** {D} {Cosine distance}; $N^{train},N^{test}$ {training and testing images numbers}; $V_{max},V_{min}$ {maximum and minimum speed ratios}; $V_{step}$ {Vehicle speed step-size} $d_{s}$ {Sequence length}; **Outputs:** *S* {Path-line (sequence candidate) score}; **for**
*T* ← $d_{s}$ to $N^{test}$
**do**  $M^{T}$ ← $\left[D^{T-d_{s}+1},D^{T-d_{s}+2},\cdots ,D^{T}\right]$; // Local searching matrix.  *j* ← 1; // Path number (sequence candidates number) initialization.  **for**
*s* ← 0 to $\left(N^{train}-V_{max}\times d_{s}\right)$
**do**   **for**
*V* ← $V_{min}$: $V_{step}$: $V_{max}$
**do**    $S_{T,j}$ ← 0;    **for**
*t* ← $\left(T-d_{s}+1\right)$ to *T*
**do**     $k\left(t\right)\leftarrow s+V(t-\left(T-d_{s}+1\right))$; // *k* is a line index in the column vector $D^{t}$; *s* is the training image number where the path originated in.     $S_{T,j}$ ← $S_{T,j}+D_{k(t)}^{t}$; // Score S is calculated for each possible path.    **end for**    *j* ← j+1; Sequence candidate number updating.   **end for**  **end for**  $SS_{T}=\frac{\min_{j}\left\{S_{T,j}\right\}}{\min_{j(j\neq m1)}\left\{S_{T,j}\right\}}$; m1 is the index of minimum score.  if $SS_{T}<=Th$  Matching validation is positive;  Vehicle position ← The matched training image position  if $SS_{T}>Th$  Matching validation is negative;  Vehicle position ← NaN (no position results) **end for**

## 4. Experimental Setup

### 4.1. Experimental Platform

**Figure 4:**
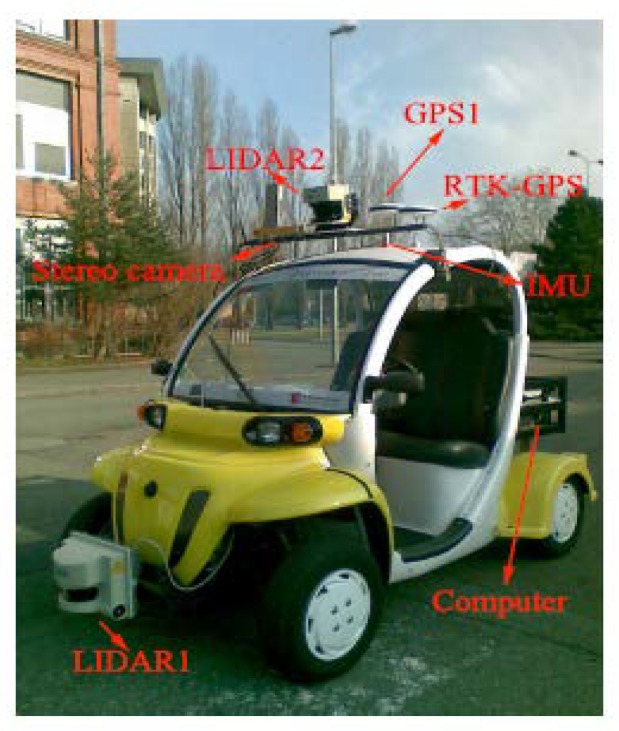
The experimental vehicle equipped with sensors (camera and RTK-GPS).

To evaluate the effectiveness of our approach, the proposed approach tests two changing data sets: one is our own and the other is an open public data set. Our data were acquired by an experimental GEM (Global Electric Motorcars) vehicle equipped with a stereoscopic Bumblebee XB3 camera system (16 Hz, 1280 × 960 image size), a RTK-GPS receiver (10 Hz), and two SICK LMS221 laser range finders, as shown in Figure 4.

### 4.2. Data Sets and Ground Truth

Four data sets with different characteristics (as described in Table 2) will be used to evaluate our method.

(1) UTBM-1 data set

**Figure 5:**
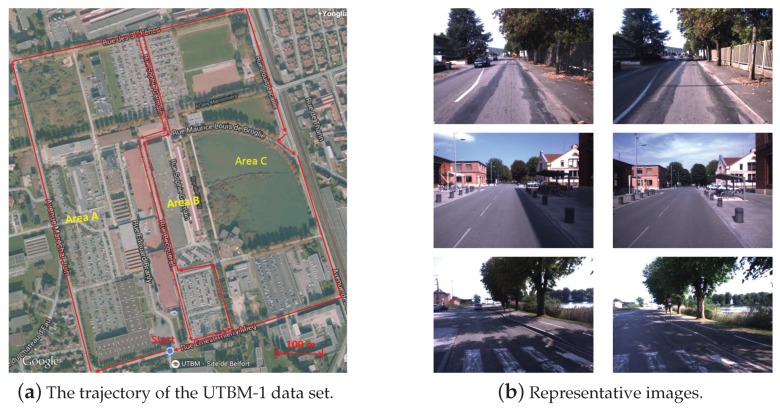
The trajectory of the UTBM-1 data set and its representative images: (**a**) The trajectory acrosses forest, city, and parking areas. (**b**) Three representative examples of appearance and shadow variations. The images in each row are taken in the same place at different times (interval time of one week).

In the UTBM-1 data set, the experimental traversed about 4 km in an urban area. As illustrated in Figure 5a, the driving trajectory crossed three typical areas: urban city (area A), factory district (area B) and a natural scene place (area C). Some representative examples of the UTBM-1 data set are shown in Figure 5b. From this figure, the changing of shadow, vegetation, and field of view between the testing and training images can be also seen. The training and testing data were collected respectively on 11 September 2014 and 5 September 2014. Among all the acquired images (at about 16 Hz), only a subset of images were selected to perform matching between the training and testing data sets (848 images for training trajectory and 819 images for testing trajectory). The average distance interval between two selected frames was around 3.5 m. To tag the reference images (training database), each image was associated with its GPS position obtained by an RTK-GPS receiver.

(2) UTBM-2 data set

**Figure 6:**
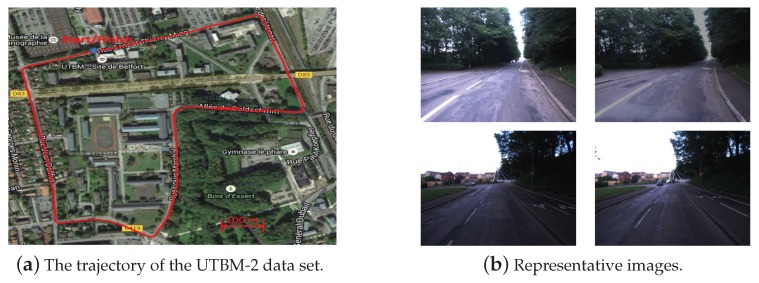
The trajectory of the UTBM-2 data set and its representative images: (**a**) The trajectory acrosses forest, city, and parking areas. (**b**) Two representative examples of illumination variations. The images in each row were taken in the same place at different times (morning vs. afternoon).

The data set UTBM-2 was collected on 5 September 2014 when the vehicle traversed on a 2.3 km long route in the urban city of Belfort. The trajectory and some image examples can be seen in Figure 6. The two traversals of this data set were conducted in the morning and afternoon. As shown in Figure 6b, there was a huge illumination variation between the images of training and testing. A total of 1060 images were used for the twice traveling (540 and 520 images for two traversals respectively).

(3) Nordland data set

**Figure 7:**
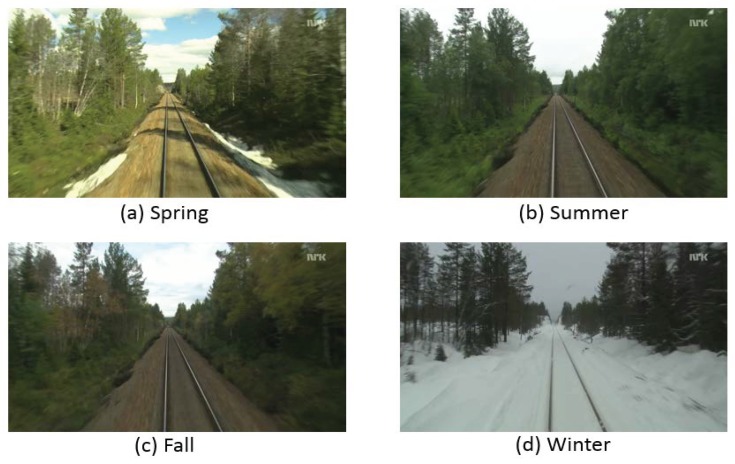
Four representative examples of the Nordland data set (each image corresponds to a different season).

The Nordland data set has four video footages of a 728-km long train ride taken in northern Norway in four seasons [37]. As demonstrated in Figure 7, there was a huge appearance variation between the four seasons due to seasonal changing. The different landscape (plants and mountains) and local weather conditions (i.e., sunshine, clouds, rain, and snowfall) were experienced on the long trip. The original videos were recorded at 25 fps with a resolution of 1920 × 1080. GPS readings were recorded in conjunction with the video at 1 Hz. The full-HD recordings have been time-synchronized such that the position of the train in an arbitrary frame from one video correspond to the same frame in any of the other three videos. In our experiment, frames were extracted from the original videos at 0.1 fps.

(4) City Center data set

**Figure 8:**
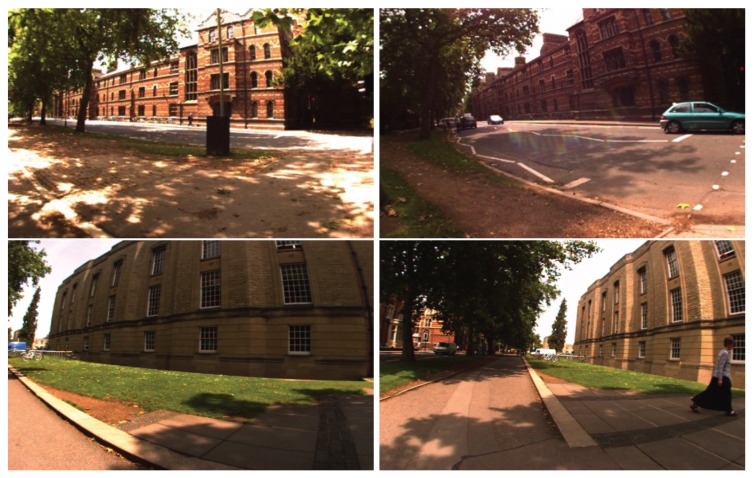
City Center data set [38]: twice traveling. The left column shows the training images, and the right column shows the testing images.

The City Center data set was collected by Mobile Robotics Group of the University of Oxford [38]. The robot traveled twice in one day around a loop with a total path length of 2 km; a total of 2474 images were collected while traveling. This data set was collected on a windy day with bright sunshine, which makes the abundant foliage and shadow features unstable, as can be observed in Figure 8.

For all four data sets, the ground truth was constructed by manually finding pairs of frame correspondences based on the GPS position. A match is considered as a true positive when it is within 1∼3 frames of the ground truth (depending on the frame rate of the recorded data set), otherwise the match is considered a false positive.

### 4.3. Performance Evaluation

Precision-recall characteristics are widely used to evaluate image retrieval abilities. Therefore, our evaluation methodology is based on precision-recall curves and $F_{1}$ scores. The final curve is computed by varying the threshold Th in a linear distribution between 0 and 1 and by calculating the corresponding values of precision and recall.

In our experiments, the training image number is larger than or equal to the testing images number; thus, each testing image has a ground-truth match. Therefore, among the positives, there are only true positives (correct results among successfully validated image matching candidates) and false positives (wrong results among successfully validated image matching candidates). The sum of the true positives and false positives is the total retrieved images number.

More specifically, precision is the ratio of true-positives over the retrieved image numbers (the number of all the successfully validated image matching candidates), and recall is the ratio of true-positives over the total testing images. One hundred threshold values are processed to obtain well-defined curves. A perfect system would return a result where both precision and recall have a value of one. Based on the precision and recall, $F_{1}$ score can be defined as

(8) $$F_{1}=2\times \frac{precision\times recall}{precision+recall}$$

## 5. Experimental Results

### 5.1. Performance Comparison between Single Images and Sequences Bsed Approach

Traditionally, visual localization has been performed by considering places as single images. However, other more recent proposals, such as SeqSLAM, changed this concept and introduced the idea of recognizing places as sequences of images.

**Figure 9:**
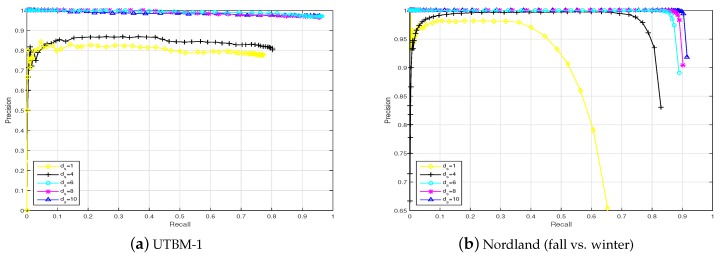
Two examples of a performance comparison of our proposal depending on the image sequence length ($d_{s}$) in the challenging UTBM-1 and Nordland data sets (fall vs. winter). The feature used here is the conv4 layer.

In this section, the place recognition performances based on sequences of images and single images are compared. In Figure 9, the results obtained for the UTBM-1 and Nordland data sets are presented. Attending to the precision-recall curves depicted in Figure 9, the influence of the sequence length ($d_{s}$) is decisive to improving the performance of visual localization in life-long conditions. It can be clearly found that the approach using sequences allows for better results than those of a single image (almost no recall at 100% precision) to be achieved in long-term visual localization.

Furthermore, there is a limit near a length of 8 for the UTBM-1 data set and a length of 6 for the Nordland data set from which the results are not greatly enhanced. Based on this sequence length comparison and the driving speed, a sequence length of $d_{s}=8$ was chosen for data sets UTBM-1 and UTBM-2 in the rest of the experiments and results. For the City Center data set, the sequence length was set to 3, and that for the Nordland data set was 6. For all data sets, the speed ratio limits were $V_{max}$ = 1.1 and $V_{min}$ = 0.9, and a step size of $V_{step}$ = 0.04 was set according to the experiment tests.

**Figure 10:**
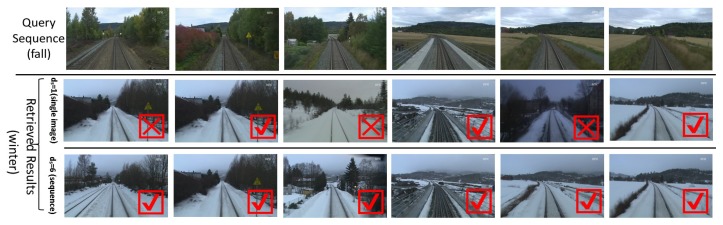
Frame match examples from the Nordland (fall vs. winter) data sets. The top row shows a query sequence, and the middle and third rows show the frames recalled by $d_{s}=1$ (single image) and $d_{s}=6$, respectively. Visual recognition based on sequence matching achieves a better performance than that of a single image.

Figure 10 shows frame match examples on the Nordland data set (fall vs. winter). Despite the large appearance variations between different seasons, the proposed ConvNet-based visual localization using sequence matching attained better recognition results that those obtained using a single image.

### 5.2. Comparison of ConvNet Features Layer-By-Layer

This section provides a thorough investigation of the utility of different layers in the ConvNet hierarchy for place recognition and evaluates their individual robustness against the two main challenges in visual place recognition: appearance and illumination changes.

#### 5.2.1. Appearance Change Robustness

**Figure 11:**
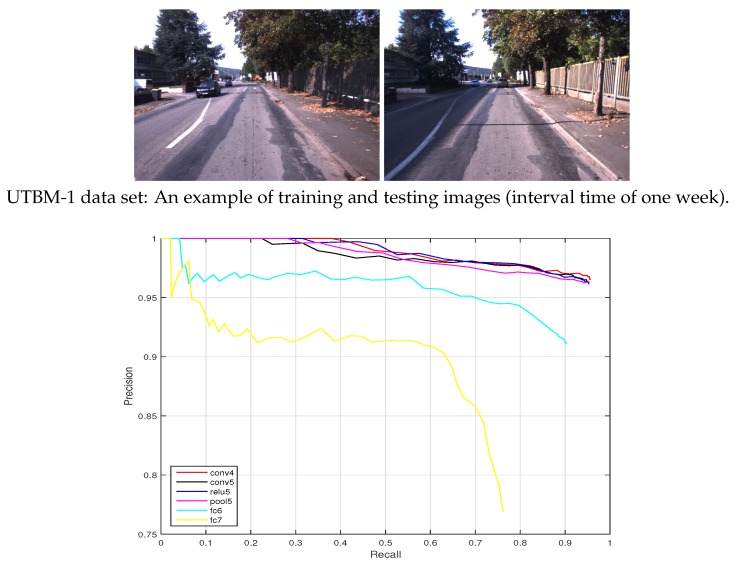
Precision-recall curves for the UTBM-1 data set (the trajectory acrosses forest, city, and parking areas) ($d_{s}=8$).

**Figure 12:**
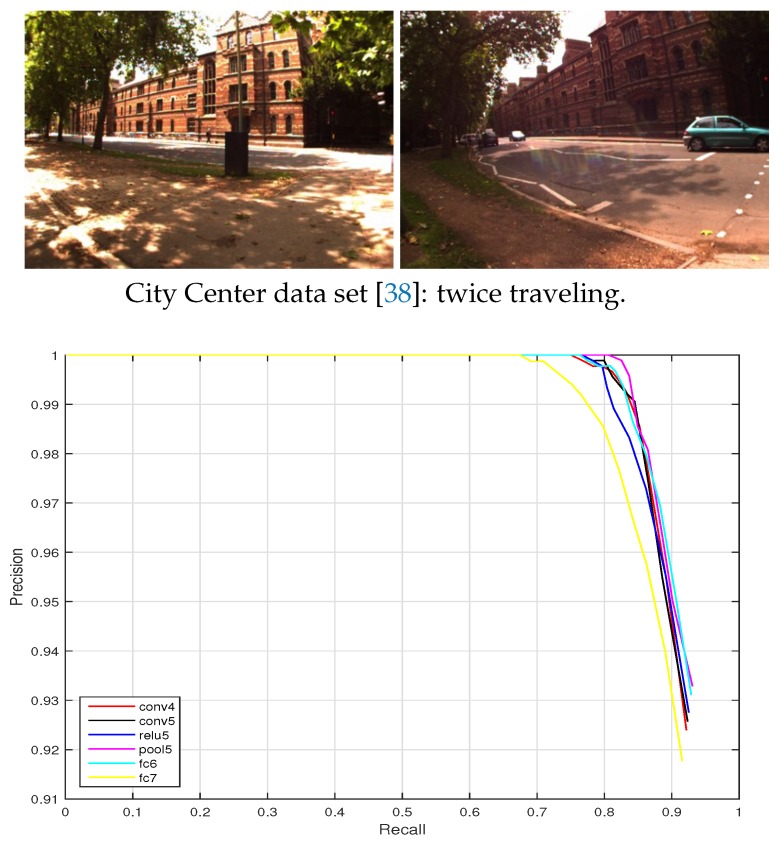
Precision-recall curves for the City Center data set (the trajectory acrosses city and parking areas) ($d_{s}=3$).

**Figure 13:**
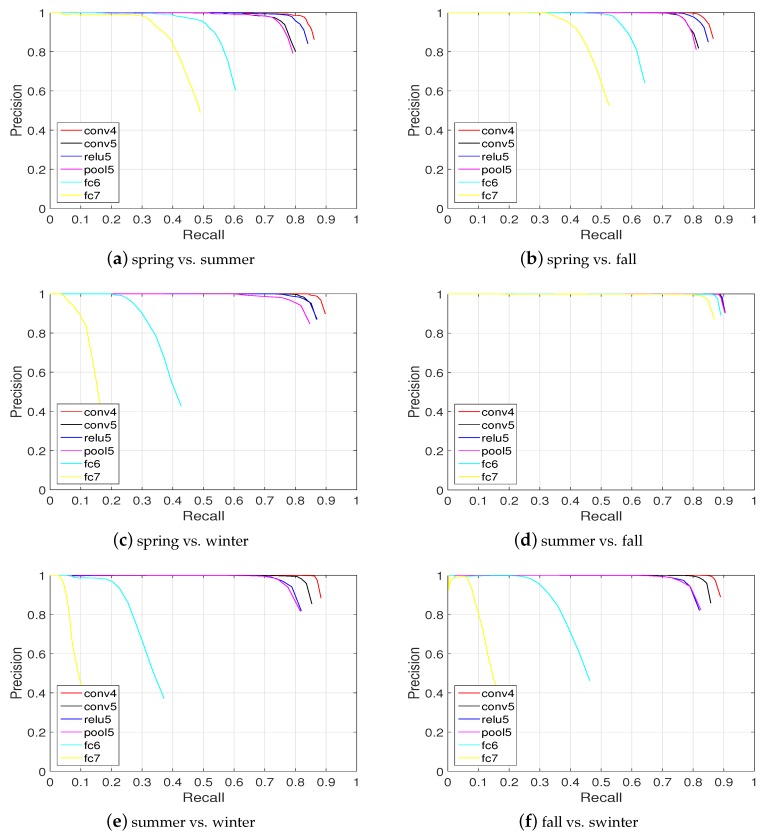
Place recognition across seasons on the Nordland data set. It can be seen that conv4 and conv5 perform better than the others, while fc6 and fc7 are the worst ($d_{s}=6$).

(1) UTBM-1 data set: The interval time of training and testing data collection was one week. As illustrated in Figure 11 (top), the appearance between test and training images has minor changes and the viewpoint has medium variations. The precision-recall curves of place recognition are shown in Figure 11 (bottom). It can be seen that a recall obtained for the conv4 layer at a totally correct level is around 40%. While the performances of layers fc6 and fc7 are poor.

(2) City Center data set: This data set was acquired along public roads near the oxford city center with many dynamic objects such as traffic and pedestrians. The precision-recall curves are shown in Figure 12. Except that the recall at 100% precision of the layer fc7 is around 70%, the performance of the other layers (conv4, conv5, relu5, pool5, and fc6) reaches above a 75% recall at a totally correct level. The conv4 layer is the best one, achieving the highest recall level.

(3) Nordland data set: It is probably the longest (3000 km) that can be currently used for a life-long visual topological localization evaluation. It contains four videos with very strong seasonal appearance changes as depicted in Figure 7. The precision-recall curves of different cases (season for training vs. season for testing) are reported in Figure 13.

It can be seen that in the case of summer vs. fall, the performances obtained from the six layers (conv4, conv5, relu5, pool5, fc6, and fc7) are excellent (around 80% recall at the 100% precision level). For the other cases, conv4, conv5, relu5, and pool5 are more robust than the higher layers fc6 and fc7 considering appearance changes.

Figure 11, Figure 12 and Figure 13 show the resulting precision-recall curves for appearance changing situations. The mid-level features from layers conv4 and relu6 are more robust against appearance changes than features from the other layers. Especially in the Nordland data set, the conv4 layer feature achieves above an 85% recall at the 100% correct level of all the tested cases. While the higher layers (i.e., fc6 and fc7) in the feature hierarchy lack robustness and exhibit an inferior place recognition performance.

#### 5.2.2. Illumination Change Robustness

**Figure 14:**
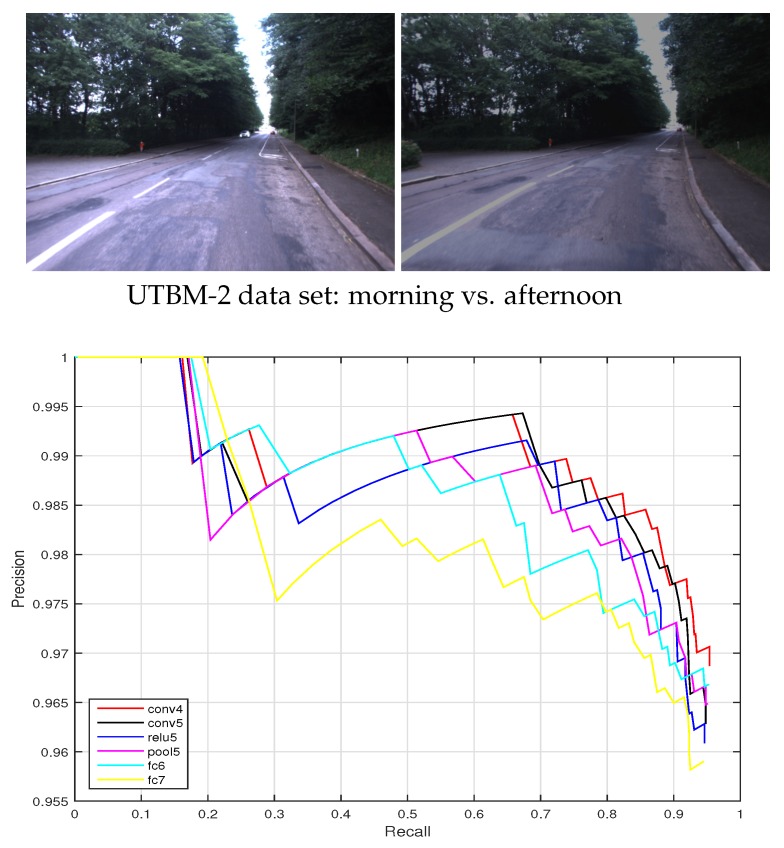
Precision-recall curves for the UTBM-2 data set considering different ConvNet layers ($d_{s}=8$).

Since illumination is another important influence factor in visual recognition, we investigate the ConvNet feature performances on the UTBM-2 data set that considers an illumination variation between morning and afternoon. The precision-recall curves are presented in Figure 14. As can be seen in Figure 14, the recall of ConvNet features from layer conv4 achieved is around 40%, which performs better than the other layer features (conv5, relu5, pool5, fc6, and fc7) to deal with the severe illumination changes. Since pretrained networks model were trained with good quality images, some layers do not show strong robust abilities in illumination variance situations.

Table 3 shows $F_{1}$ scores obtained for different layers and other state-of-the-art methods like SeqSLAM and FAB-MAP. For a SeqSLAM comparison, the OpenSeqSLAM code [20] was used and the same sequence lengths were taken as settled above. While the other parameters are set to default values as reported in Reference [20]. For a FAB-MAP comparison, the OpenFABMAP code [39] was used.

By comparing our approach to the SeqSLAM method under extreme appearance changes in Table 3, it can be found that the obtained $F_{1}$ scores (the harmonic average of the precision and recall) based on ConvNet features extracted by layer conv4 are above 0.85, which exceeds the SeqSLAM and FAB-MAP performances. In addition, it can be noticed that the performance of the ConvNet feature extracted from the conv4 layer is better than the other layers, especially the higher layers (i.e., fc6 and fc7). It confirmed again that the middle layers such as conv4 contain more useful image information due to advantages of the spatial information from low layers and semantic information from high layers being taken.

### 5.3. Local Sensitive Hashing for Real-Time Place Recognition

In contrast to typical computer vision benchmarks where the recognition accuracy is the most important performance metric, visual localization for vehicles or robots always needs agile algorithms for real-time application [40]. In the above studies, conv4 has shown its strong ability in place recognition. However, computing the cosine distance between many 64,896 dimensional conv4s is an expensive operation since Locality Sensitive Hashing (LSH) is arguably the most popular feature compression method and widely used in the field of information retrieval and computer vision [41]. For a fast place recognition, the LSH method is used, which maps the conv4 feature $f_{conv4}$ to a low-dimensional binary vector:

(9) $$H\left(K\right)=sign\left(w^{⊤}f_{conv4}+b\right)$$

where *w* is a *K* dimension data-independent random matrix, which satisfies a standard Gaussian distribution [41] and *b* is a random intercept. In our experiment, the conv4 feature $f_{conv4}$ is normalized with a zero mean; then, an approximately balanced partition is obtained with *b* = 0. Thus, the high dimension feature is converted into a low *K* dimension binary bits. The binary bit vectors can then be compared using the hamming distance more efficiently.

**Figure 15:**
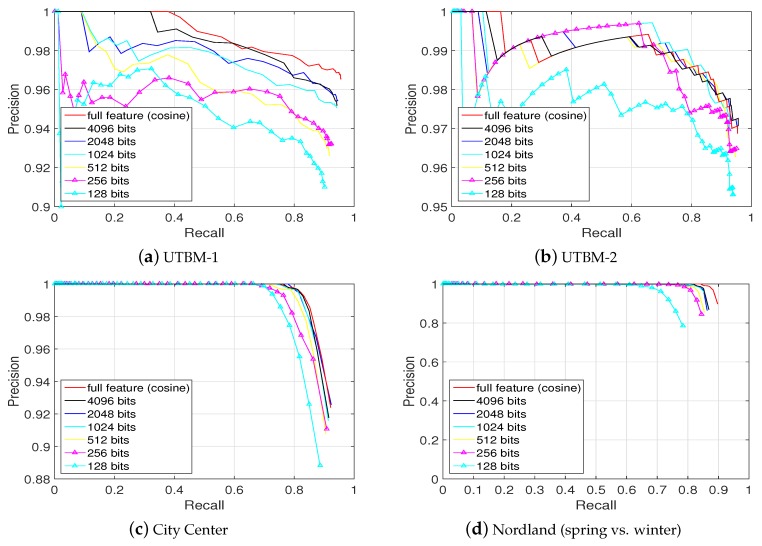
Precision-recall curves of different hash bit lengths. The cosine distance over the full feature vector of 64,896 dimensions (red) can be closely approximated by the hamming distance over bit vectors of length 4096 (dark) without losing much performance. This corresponds to a compression of 63.1%.

In Figure 15, the place recognition performance achieved with the hashed conv4 feature vectors of different hash bit lengths ($2^{8}$ *…* $2^{12}$ bits) on the four data sets is compared. Hashing the original 64,896 dimensional vectors into 4096 hash bits corresponds to a data compression of 63.1%. In addition, the 4096-hash-bit representation retains approximately 95% of the original place recognition performance. It can be seen that, from Figure 15, when the length of hash bits is decreasing, the place recognition performance is also descending.

Table 4 shows the $F_{1}$ scores of different hash bit lengths achieved in four data sets. The average times per matching are also presented. The experiments are conducted on a laptop machine with intel i7-4700MQ CPU and 32 Gb RAM.

As shown in Table 4, the average time per matching using 4096 hash bits is 0.0291 s which corresponds almost to a speed-up factor of 12 compared to using the cosine distance over the original conv4 feature requiring 0.3259 s per matching. Compared with the full feature matching, using the 4096 hash bits representing the original full feature permits the matching to be faster and easier. There is no doubt that, for larger scale data sets, the speed-up advantages can be more significant.

### 5.4. Visual Localization Results

**Figure 16:**
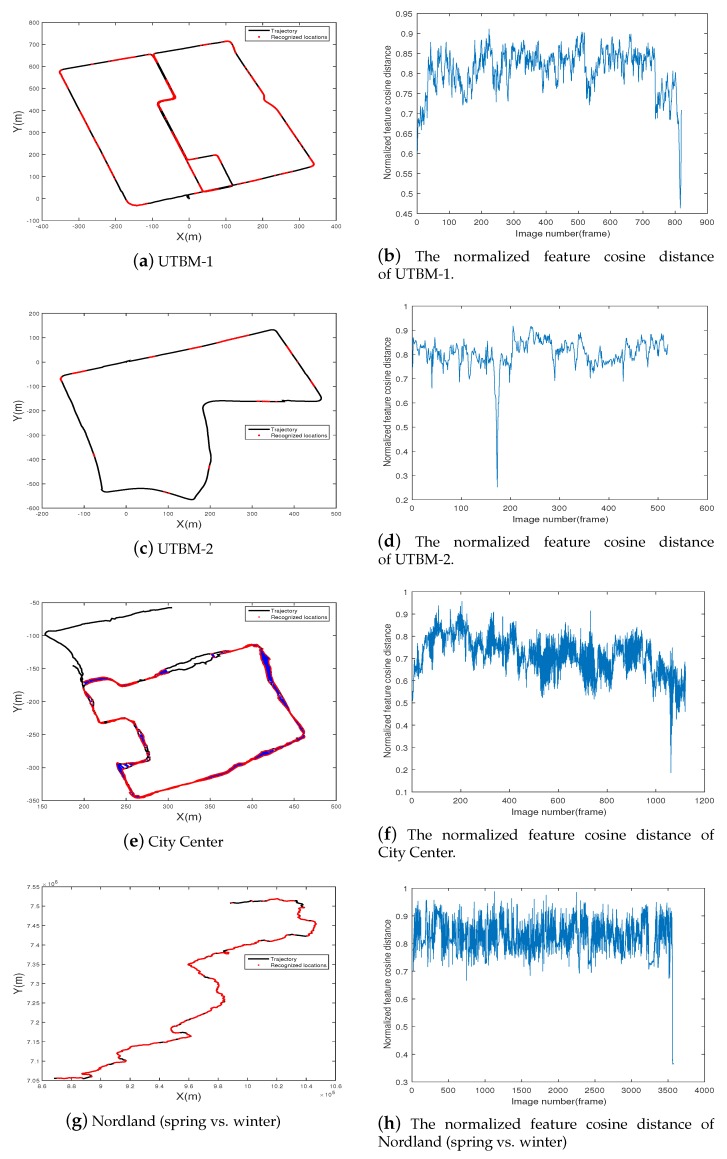
The visual localization results in the four data sets. The used feature is 4096 hash bits of the conv4 layer. In the left column, two images from the same location (on the basis of appearance alone) are marked with red points and joined with a blue line. In the right column are the corresponding normalized feature cosine distances.

For visual localization based on place recognition, the recognition rate at a high precision level is a key indicator in reflecting whether the system is robust enough to determine the position under a changing environment. A correct place recognition means a successful visual localization, while an incorrect place recognition could cause a huge localization error. Therefore, the higher the recognition rate at a 100% precision is, the more robust the visual localization system is. Figure 16 shows the final place-recognition-based visual localization results for the different data sets at a precision level of 100%. Regardless of the appearance and illumination changes, the proposed method can still localize the vehicle in most places.

In Table 5, recall ratios at different precision levels are given. Using 4096 hash bits at 100% precision, the proposed approach achieves above a 75% recall on the City Center data set and above 72.88% on the more challenging Nordland data set (Spring vs. Winter), while on the UTBM-1 and UTBM-2 data sets, the recall are 32.88% and 11.54% respectively.

## 6. Conclusions and Future Works

In this paper, a visual vehicle localization approach based on ConvNet features and localized sequence matching is proposed. The approach takes advantages of the ConvNet image representation ability and localized sequence matching, which make place recognition fast and accurate. We also compared the proposed approach with state-of-the-art methods (SeqSLAM and FAB-MAP) on four typical data sets that consider big challenges in visual place recognition: appearance and illumination changes. The experimental results showed that ConvNet feature conv4 can achieve a good performance with above a 0.89 $F_{1}$ score. In addition, for satisfying real-time constraints, the speed-up approach based on the LSH method was used to compress the high dimension of ConvNet features. By using the 4096 hashing bits representation to replace the original conv4 feature, each matching process was almost 12 times faster. The proposed visual localization approach allowed the vehicle to localize itself in changing environments.

In future work, we will extend our understanding of ConvNet features and explore how to use transfer learning to service the place recognition. Additionally, we will also considering how to train a specific end-to-end CNN for a life-long visual localization under changing conditions.

## Figures and Tables

**Table 1:** The layers from the caffe-alex ConvNet model used in our evaluation and their output dimensionality (height × width × feature map number).

Layer	Dimensions	Layer	Dimensions
conv4	13 × 13 × 384	fc6	1 × 1 × 4096
conv5	13 × 13 × 256	fc7	1 × 1 × 4096
relu5	13 × 13 × 256		
pool5	6 × 6 × 256		

**Table 2:** Descriptions of the main characteristics of the data sets employed in the experiments.

Data Set	Length	No. Images	Description
UTBM-1	2 × 4.0 KM	training: 848; testing: 819	minor variations in appearance and illumination
UTBM-2	2 × 2.3 KM	training: 540; testing: 520	medium variations in appearance and illumination
Nordland	4 × 728 KM	4 × 3568	severe variations in appearance
City Center	2 × 2.0 KM	2 × 1237	medium variations in viewpoint

**Table 3:** $F_{1}$-Scores considering different Caffe-AlexNet layers and other state-of-the-art methods (SeqSLAM and FAB-MAP). The † means the $F_{1}$ score is smaller than 0.01, and ‡ means experiment fails. For each data set, the highest $F_{1}$-Score is bold.

Dateset		Caffe-Alex Layers						SeqSLAM	FAB-MAP
		conv4	conv5	relu5	pool5	fc6	fc7		
Norland	spring vs. summer	**0.8967**	0.8427	0.8734	0.8354	0.6722	0.5455	0.7222	‡
	spring vs. fall	**0.8984**	0.8572	0.8821	0.8579	0.7098	0.5859	0.7015	‡
	spring vs. winter	**0.9255**	0.8987	0.8983	0.8750	0.4795	0.2387	0.6685	‡
	summer vs. fall	**0.9396**	0.9381	0.9388	0.9375	0.9286	0.9047	0.6960	‡
	summer vs. winter	**0.9245**	0.8935	0.8581	0.8497	0.4142	0.1817	0.5117	‡
	fall vs. winter	**0.9288**	0.8922	0.8598	0.8599	0.5119	0.2337	0.5293	‡
UTBM-1		**0.9607**	0.9576	0.9576	0.9583	0.9607	0.7762	0.7222	0.2356
UTBM-2		**0.9622**	0.9564	0.9544	0.9574	0.9593	0.9516	0.7180	0.4813
City Center		0.9288	0.9246	0.9264	**0.9317**	0.9299	0.9166	†	0.5326

**Table 4:** $F_{1}$-scores and matching time comparison of different lengths of hash bits. For each data set, the highest $F_{1}$-Score is bold.

Method	$F_{1}$ Scores				Average Time Per Matching (All Data Sets)
	UTBM-1	UTBM-2	City Center	Nordland (Spring vs. Winter)	
256 bits	0.9411	0.9574	0.9094	0.8817	0.0135 s
512 bits	0.9478	0.9554	0.9084	0.8944	0.0147 s
1024 bits	0.9460	0.9612	0.9162	0.9046	0.0170 s
2048 bits	0.9521	0.9632	0.9246	0.9064	0.0209 s
4096 bits	0.9521	**0.9641**	0.9166	0.9099	0.0291 s
Full feature (conv4)	**0.9607**	0.9622	**0.9228**	**0.9255**	0.3259 s

**Table 5:** The recall results at 100% precision. The † means the accuracy is smaller than 10%.

Dateset		Recall Results at 100% Precision			
		Full Feature (conv4)	4096 Hash Bits	FAB-MAP	SeqSLAM
Norland	spring vs. summer	57.09	69.02	†	45.71
	spring vvs. fall	64.66	67.26	†	33.91
	spring vs. winter	76.77	72.88	†	35.53
	summer vs. fall	86.88	87.67	†	47.89
	summer vs. winter	60.47	28.26	†	22.84
	fall vs. winter	82.16	79.65	†	15.82
UTBM-1		37.97	32.88	†	20.16
UTBM-2		16.35	11.54	†	8.53
City Center		75.04	76.29	31.78	52.63

## Abbreviations

The following abbreviations are used in this manuscript:

ConvNet	Convolutional Network
LSH	locality sensitive hashing
FAB-MAP	Fast Appearance Based Mapping
SeqSLAM	Sequence Simultaneous Localisation and Mapping
